# Impact on oral health-quality of life in infants: Multicenter study in Latin American countries

**DOI:** 10.1590/0103-6440202204929

**Published:** 2022-04-29

**Authors:** Saul Martins Paiva, Letícia Pereira Martins, Jéssica Madeira Bittencourt, Licet Alvarez, Ana Maria Acevedo, Verónica Cepeda, Carmen Aminta Galvez, Cassia Gaberllini, Sylvia Gudiño, Stefania Martignon, Vidal Pérez, Olga Zambrano, Diana Zelada, Rita S. Villena, Pablo Salgado, Aldo Squassi, Noemi E. Bordoni

**Affiliations:** 1 Universidade Federal de Minas Gerais - UFMG, Department of Pediatric Dentistry, Belo Horizonte, MG, Brasil.; 2 Universidad de la República Uruguay - UDELAR, Department of Pediatric Dentistry. Montevideo, Uruguay; 3 Universidad Central de Venezuela, Dentistry Faculty, Dental Research Institute, Caracas, Venezuela; 4 Universidad Internacional del Ecuador(UIDE), Escuela de Odontología. Quito, Ecuador; 5 Ministerio de Salud de Panamá, Panamá.; 6 Universidade Estadual de Londrina- UEL, Children´s Especiality Clinic Director, Bebê Clinica. Londrina, PR, Brasil; 7 Universidad de Costa Rica. Pediatric Dentistry Master, San José Postgraduate Study Sistem. Costa Rica; 8 Universidad El Bosque- UEB, Profesor, ÚNICA - Caries Research Unit, Research Department, Bogotá, Colombia; 9 Universidad de Talca, Department of Pediatric Dentistry. Talca, Chile.; 10 Universidad del Zulia, Maracaibo- Zulia, Venezuela.; 11 Universidad San Martin de Porres - USMP, Department of Pediatric Dentistry. Lima, Perú.; 12 Universidad de Buenos Aires - UBA, Research in Public Health Institute, Buenos Aires, Argentina

**Keywords:** Quality of life, oral health, health-related quality of life, child, epidemiology

## Abstract

To assess the impact of oral conditions on oral health-related quality of life (OHRQoL) in infants in ten Latin America countries (LAC). A cross-sectional study was conducted with 930 pairs of 1-to-3-year-old children/parents from 10 LAC, as a complementary study of the Research Observatory for Dental Caries of the Latin American Region. The scale ECOHIS, previously tested and valid in ten countries, was applied to parents/caregivers of children to measure OHRQoL. Statistical analysis included descriptive data analysis and one-way analysis of variance (ANOVA-One-Way) were performed to compare age groups with OHRQoL. Bootstrapping procedures (1000 re-samplings; 95%CI Bca) were performed. The mean scores of the ‘Child Impact’ section in the LAC was 4.0(±8.3), in the ‘Family Impact’ section was 2.0(±4.0), and in overall ECOHIS score was 6.0(±12.0). In the ‘Child Impact’ section, Argentina 10.0(+2.4) and Venezuela 17.8(±17.5) demonstrated mean scores higher than the LAC total data. In the ‘Family Impact’ section, the countries with higher mean scores were Argentina 4.9(±2.0), Ecuador 2.1(±3.1) and Venezuela 7.9(±7.8). In the overall ECOHIS score, Argentina 15.1 (±4.1) and Venezuela 25.7(±25.2) has higher mean scores than the values of LAC. There is an association between children's age and parents' report of impact on the OHRQoL (p<0.001). Three-year-olds had a higher mean when compared to one- and two-year-olds, both in the Impact on the Child and Impact on the Family (p<0.001) sections, as well as in the overall ECOHIS (p<0.001). In conclusion, there are differences in OHRQoL among Latin American countries, impacting older children more significantly.

## Resumo

Avaliar o impacto das condições bucais na qualidade de vida relacionada à saúde bucal (QVRSB) em crianças de dez países da América Latina (AL). Foi realizado um estudo transversal com 930 pares de crianças/pais de 1 a 3 anos de 10 países da AL, como estudo complementar do *Research Observatory for Dental Caries of the Latin American Region*. A escala ECOHIS, previamente testada e validada em dez países, foi aplicada a pais/cuidadores de crianças para mensurar a QVRSB. A análise estatística incluiu análise descritiva de dados e análise de variância unidirecional (ANOVA-One-Way) para comparar grupos etários com QVRSB. Procedimentos de bootstrapping (1000 reamostragens; 95%IC Bca) foram realizados. A pontuação média da seção 'Impacto na Criança' na AL foi 4,0 (±8,3), na seção ‘Impacto na Família’ foi 2,0 (±4,0) e no escore total do ECOHIS foi 6,0 (±12,0). Na seção 'Impacto na Criança', Argentina 10,0(+2,4) e Venezuela 17,8(±17,5) demonstraram pontuações médias superiores aos dados totais da AL. Na seção ‘Impacto na Família’, os países com pontuações médias mais altas foram Argentina 4,9(±2,0), Equador 2,1(±3,1) e Venezuela 7,9(±7,8). No escore total do ECOHIS, Argentina 15,1 (±4,1) e Venezuela 25,7(±25,2) apresentaram escores médios superiores aos valores de AL. Houve associação entre a idade das crianças e o relato dos pais de impacto na QVRSB (p<0,001). As crianças de três anos tiveram média maior quando comparadas às de um e dois anos, tanto nas seções 'Impacto na Criança' e ‘Impacto na Família’ (p<0,001), quanto no escore total ECOHIS (p<0,001). Em conclusão, houveram diferenças na QVRSB entre os países da América Latina, impactando de forma mais significativa as crianças mais velhas.

## Introduction

Over the past decades, the use of patient-centered outcome measures (PROMs) has become common in dentistry due to the need to incorporate patient-reported measures along with normative criteria defined by the dentist, as oral health is a multidimensional concept ^1^. PROMs are identifiable, valid, and reliable instruments that aim to assess a patient's health status through the patient's own perception ^1^. Thus, provides healthcare professionals with information beyond the clinical only assessment, thus taking a holistic view of the patient and family ^2^. Self-reported outcomes are of pivotal importance to planning public and individual oral health care, contributing to the improvement of oral health through disease prevention and health promotion programs ^3^. The most studied patient-centered outcome in dentistry is the oral health-related quality of life (OHRQoL).

The term “quality of life” was defined as a multidimensional concept integrating all areas of life and referring to both objective conditions and subjective components. There are various conceptual models of “quality of life” proposed by different authors ^4^ ^,^ ^5^. OHRQoL is a subjective construct, which aims to measure the broad consequences of oral conditions on the individual's well-being and daily life. It is a dynamic construct, that can be impacted by the social, cultural, and political context in which the individual is inserted ^3^ ^,^ ^6^ ^,^ ^7^. This construct provides important information for the dentists about the decision-making process and prioritization of oral health care system ^8^. In addition, it is important to act in the allocation of resources, development and evaluation of public health policies ^8^.

Despite the importance of incorporating patient-reported outcome measures, both in clinical practice and in the scientific field, there are few studies on the impact of oral conditions on OHRQoL in infants of Latin American countries (LAC) ^9^ ^,^ ^10^ ^,^ ^11^ ^,^ ^12^ ^,^ ^13^ ^,^ ^14^ ^,^ ^15^. The studies with infants have important limitation that must be recognized. Many of them have small samples of institutionalized or clinical-based children. 

Multicenter studies on OHRQoL with the use of standardized and validated instruments, encompassing several countries of a geographic region, such as Latin America region, are of pivotal importance to guarantee a broad panorama of the perception of parents/caregivers about the impact of oral health on children's quality of live. Thus, it will be possible to make comparisons and define the main priorities of each country and of the region, as well as guiding public health care systems and health professional approach in order to make decisions on actions and programs of prevention and health promotion ^16^. It is important to emphasize that to carry out studies on OHRQoL in this age group is necessary to use proxy-reported instruments, as children under the age of three are not able to provide valid and reliable information on their OHRQoL ^17^.

Therefore, this study aims to assess the impact of oral conditions on OHRQoL in infants aged 1 to 3 years and their families in ten LAC. The hypothesis is that there is a negative impact of oral conditions on the OHRQoL of infants.

## Material and methods

The present study conforms to guidelines from the Strengthening the Reporting of Observational studies in Epidemiology (STROBE Statement) ^18^.

### Ethical requirements

This multicenter study was approved by the Ethics and Research Committee of the Facultad de Odontología de la Universidad San Martin de Porres (USMP), Lima, Peru, with Act No. 08 of December 12, 2017 and Committees of the co-participating Universities. This study was conducted in accordance with the principles expressed in the Declaration of Helsinki (revised in World Medical Association 2013). Parents/caregivers signed an informed consent form and were informed about the objectives, importance, and methodology of the study.

### Study design and eligibility criteria

This cross-sectional study was carried out as a complementary arm of the Research Observatory for Dental Caries of the Latin American Region (OICAL), which is a project of the Regional Development Program of the International Association for Dental Research (IADR RDP LARRDP-LAR/IADR). LAC is a region of the American continent, with more than 596 million inhabitants (http://latinoamericana.wiki.br/) and a territory of approximately 19,200,000 km². Representatives of 10 IADR Divisions and Sections of Latin American countries (Argentina, Brazil, Chile, Colombia, Costa Rica, Ecuador, Panama, Peru, Uruguay and Venezuela), 12 dental schools, and the Ministry of Health of Panama, met in Lima, Peru, in 2018. This meeting aimed to carrying out theoretical and clinical training to standardize criteria for collecting data on oral health-reported quality of life for children and adolescents.

The sample of this study consisted of children aged one to three years from nursery and public preschools the ten LAC participating in the OICAL Project. In each country, a city was selected by for convenience (8 capital cities and 2 large urban cities) based on the place of work of each representative. Data collection was performed from August 2018 to March 2019. 

The inclusion criteria were parents/caregivers of male and female children aged one to three years, literate and capable of understanding and answering the instrument in writing based on the information provided by the questionnaire, without incorporating additional clarifications during the procedure.

### Outcome variable

OHRQoL measured using the Early Childhood Oral Health Impact Scale (ECOHIS) cross-culturally adapted and validated for use in LAC countries ^19^ ^,^ ^20^ ^,^ ^21^ ^,^ ^22^ ^,^ ^23^ ^,^ ^24^ ^,^ ^25^. ECOHIS assesses the impact of oral health conditions on the quality of life of children and their families.

The ECOHIS consists of 13 questions divided into two main parts: a "child impact" section composed of four subscales (Symptoms, Function, Psychology and Self-Image/Social Interaction) and a "family impact" section composed of two subscales (Parental Distress and Family Function). The questionnaire is scored using a five-point scale with responses ranging from "never" (score 0) to "very often" (score 4). The total score ranges from 0 to 52 and is calculated as the sum of the responses. Higher scores denote greater oral health impact or poorer OHRQoL. The “child impact" section, "family impact" section and total ECOHIS score was used in statistical analysis. The ECOHIS was self-administered in parents and it was asked to be answered by the main caregiver.

### Statistical analysis

The statistical analysis was performed using the Statistical Package for Social Sciences (SPSS for Windows, version 22.0, IBM Inc, Armonk, NY, USA). Data normality was assessed using the Kolmogorov-Smirnov tests. The assumption of homogeneity of variance was evaluated using the Levene test. Descriptive data analysis and one-way analysis of variance (ANOVA-One-Way) were performed to compare age groups with OHRQoL.

Bootstrapping procedures (1000 re-samplings; 95% CI Bca) were performed to obtain greater reliability of the results, to correct deviations from normality in the sample distribution and differences between group sizes ^26^.

Considering the heterogeneity of variance, Welch correction and post-hoc evaluation was requested using the Games-Howell technique ^27^. An a posteriori power calculation was performed, using GPower, considering an effect size of 0.10; significance level of 0.05 and a total sample size of 930 participants, reaching a power of 0.86.

## Results

A total of 930 pairs of parents/children from the ten countries participated of the study: Argentina, Brazil, Chile, Colombia, Costa Rica, Ecuador, Panama, Peru, Uruguay, and Venezuela. The percentage of male children in the total sample was of 51.9% (n=483), demonstrating a good proportion for representativeness of the population, as it covered a similar number of male and female children (Table 1).

**Table 1:** Distribution of the sample size by sex in Latin America countries

	Sex		Total sample (N%)
	Female N (%)	Male N (%)	
Argentina	48 (48.0)	52 (52.0)	100 (100.0)
Brazil	45 (41.7)	63 (58.3)	108 (100.0)
Chile	23 (51.1)	22 (48.9)	45 (100.0)
Colombia	47 (47.0)	53 (53.0)	100 (100.0)
Costa Rica	45 (45.0)	55 (55.0)	100 (100.0)
Ecuador	48 (48.0)	52 (52.0)	100 (100.0)
Panama	57 (50.9)	55 (49.5)	112 (100.0)
Peru	51 (50.5)	50 (49.5)	101 (100.0)
Uruguay	32 (47.8)	35 (52.2)	67 (100.0)
Venezuela	51 (52.6)	46 (47.4)	97 (100.0)
Latin America	447 (48.1)	483 (51.9)	930 (100.0)

The participants’ age ranged from 1 to 3 years with a mean (±SD) of 1.9 (±0.6) years. Most participants in LAC were 2 years old (60.9%), with this age group being more prevalent in Peru (93.1%), Colombia (81.0%), Costa Rica (73.0%), Argentina (67.0%), Brazil (63.9%), Uruguay (58.2%), Panama (55.4%), and Ecuador (52.0%) (Table 2).

**Figure 1:**
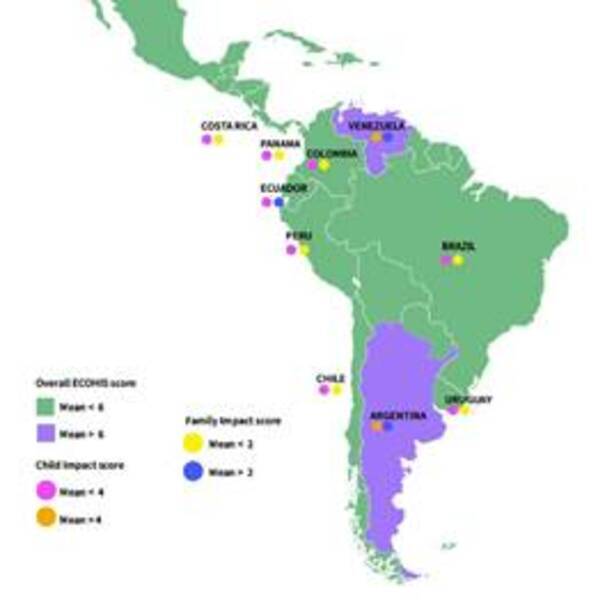
Representativeness of the mean scores of the ‘Child Impact’, ‘Family Impact’ sections and overall ECOHIS according to the mean LAC in children aged 1 to 3 years old

The mean scores of the ‘Child Impact’ section in the LAC was 4.0 (±8.3), in the ‘Family Impact’ section was 2.0 (±4.0), and in overall ECOHIS score was (6.0 ±12.0) (Table 3). In the ‘Child Impact’ section, Argentina (10.0 (+2.4) and Venezuela (17.8 ±17.5) demonstrated mean scores higher than the LAC total data. In the ‘Family Impact’ section, the countries with higher mean scores were Argentina (4.9 ±2.0), Ecuador (2.1 ±3.1) and Venezuela (7.9 ±7.8). In the overall ECOHIS score, Argentina (15.1 ±4.1) and Venezuela (25.7 ±25.2) has higher mean scores than the values ​​of LAC (Figure 1 and Table 3). Table 4 shows that there is an association between children's age and parents' report of impact on the OHRQoL (p < 0.001). Three-year-olds had a higher mean when compared to one- and two-year-olds, both in the Impact on the Child and Impact on the Family (p < 0.001) sections, as well as in the overall ECOHIS (p < 0.001).

**Table 2:** Distribution of the sample size by age in Latin America countries

	Age			Total sample (N%)
	1 year old	2 years old	3 years old	
	N (%)	N (%)	N (%)	
Argentina	13 (13.0)	67 (67.0)	20 (20.0)	100 (100.0)
Brazil	34 (31.5)	69 (63.9)	05 (4.6)	108 (100.0)
Chile	08 (17.8)	10 (22.2)	27 (60.0)	45 (100.0)
Colombia	19 (19.0)	81 (81.0)	00(0.0)	100 (100.0)
Costa Rica	27 (27.0)	73 (73.0)	00 (0.0)	100 (100.0)
Ecuador	35 (35.0)	52 (52.0)	13 (13.0)	100 (100.0)
Panama	44 (39.3)	62 (55.4)	6 (5.4)	112 (100.0)
Peru	07 (6.9)	94 (93.1)	0 (0.0)	101 (100.0)
Uruguay	23 (34.3)	39 (58.2)	5 (7.5)	67 (100.0)
Venezuela	00 (0.0)	19 (19.6)	78 (80.4)	97 (100.0)
Latin America	210 (23.7)	566 (60.9)	154 (16.6)	930 (100.0)

## Discussion

The results of the present study showed that the greatest impact of infants' oral conditions on the OHRQoL in the "impact on children" section and the general section of ECOHIS were observed in Argentina and Venezuela. In the "family impact" section, they were in Argentina, Ecuador and Venezuela. Regarding the country with the lower negative impact of oral conditions on the OHRQoL were Panama, Colombia, Costa Rica and Uruguay. It is important to emphasize that obtaining data on OHRQoL contributes to providing patient-reported outcomes, with the aim of improving the quality of pediatric dental care. In addition to contributing to the implementation of public policies aimed at minimizing social inequalities and providing better OHRQoL for children ^28^.

**Table 3:** Descriptive analyzes of the ‘Family Impact’ and ‘Child Impact’ sections, and the overall ECOHIS scores in the Latin America countries.

Countries	N	Child Impact		Family Impact		Overall ECOHIS	
		Mean	BCa 95%CI	Mean	BCa 95%CI	Mean	BCa 95%CI
		(+SD)	Mean (+SD)	(+SD)	Mean (+SD)	(+SD)	Mean (+SD)
Argentina	100	10.0 (+2.4)	9.6-10.5 (1.7-3.0)	4.9 (+2.0)	4.5-5.3 (1.5-2.5)	15.1 (+4.1)	14.4-16.0 (2.9-5.1)
Brazil	108	1.3 (+2.7)	0.8-1.8 (1.9-3.4)	0.6 (+1.7)	0.4-0.9 (0.8-2.5)	2.0 (+3.8)	1.4-2.6 (2.4-5.1)
Chile	45	1.3 (+3.7)	0.5-2.4 (0.9-6.2)	1.0 (+2.5)	0.4-1.7 (1.3-3.4)	2.3 (+5.7)	1.1-4.1 (1.6-9.1)
Colombia	100	0.8 (+2.2)	0.5-1.2 (1.4-2.8)	0.4 (+1.2)	0.2-0.6 (0.8-1.4)	1.2 (+2.7)	0.8-1.8 (1.8-3.7)
Costa Rica	100	1.4 (+3.1)	0.9-2.0 (2.1-3.9)	0.5 (+1.4)	0.2-0.8 (0.8-2.0)	1.9 (+4.2)	1.2-2.7 (2.7-5.3)
Ecuador	100	2.6 (+3.6)	1.9-3.3 (2.7-4.4)	2.1 (+3.1)	1.6-2.7 (2.6-3.6)	4.7 (+5.9)	3.5-5.7 (4.5-7.1)
Panama	112	0.34 (+1.7)	0.1-0.7 (0.7-2.4)	0.3 (+1.5)	0.1-0.6 (0.5-2.1)	0.7 (+2.7)	0.3-1.1 (1.4-3.7)
Peru	101	1.9 (+3.3)	1.4-2.5 (2.6-3.9)	1.2 (+2.4)	0.8-1.6 (1.7-3.1)	3.1 (+5.1)	2.1-4.1 (3.9-6.0)
Uruguay	67	1.2 (+2.6)	0.7-1.9 (1.5-3.4)	0.8 (+2.2)	0.3-1.3 (1.2-2.9)	1.9 (+4.5)	0.9-3.1 (2.5-6.0)
Venezuela	97	17.8 (+17.5)	14.5-21.6 (17.2-17.6)	7.9 (+7.8)	6.4-9.4 (7.6-7.9)	25.7 (+25.2)	20.6-31.0 (24.7-25.4)
Latin America*	930	4.0 (+8.3)	3.5-4.6 (7.4-9.1)	2.0 (+4.0)	1.8-2.4 (3.5-4.3)	6.0 (+12.0)	5.4-6.8.0 (10.8-13.2)

*Total data of the 10 Latin American countries: Argentina, Brazil, Chile, Colombia, Costa Rica, Ecuador, Panama, Peru, Uruguay, Venezuela. Bca95%IC= Confidence Interval Bias-Corrected and Accelerated. +SD = standard deviation.

**Table 4:** Association between children's age and parents' report of impact on OHRQoL

Age	Child Impact		Family Impact		Overall ECOHIS	
	Mean (+SD)	p-value	Mean (+SD)	p-value	Mean (+SD)	p-value
1 year old^a^	2.0 (3.8)	<0.001	1.0 (2.3	<0.001	3.0 (5.6)	<0.001
2 year old^a^	2.7 (5.2)		1.5 (3.0)		4.2 (7.9)	
3 year old^b^	11.4 (14.9)		5.2 (6.6)		18.8 (21.5)	

ANOVA-*One Way* test. Groups with different letters were statistically different (Teste post-hoc de Games-Howell with Bootstrapping (95% IC Bca)

The present study found an association between the children's age and the parents' report on the impact on the OHRQoL, with three-year-old children having a higher mean impact on the OHRQoL. This result can be explained by the fact that older children tend to have a greater number of carious lesions, as well as more severe carious lesions, which could explain a highter impact on OHRQoL ^11^. In addition, with increasing age, children increase their ability to communicate with their parents and report the impact of their oral condition on OHRQoL ^11^.

A broad overview of the perceptions of parents/caregivers of children aged 1 to 3 years in LAC promotes important data at a global level so that health professionals pay attention to the importance of the infant's initial period of life. At three years of age, a infant has had all teeth in the mouth for 1 year and, based on the data from the present study, there is mean high of negative impact on children’ OHRQoL in LAC. This mean/frequency high was also found in previous studies from several LAC countries, with parents reporting a higher impact on the Child Impact section than on the Family Impact section ^11^ ^,^ ^12^ ^,^ ^15^. In addition, LAC is mainly composed of low- and middle-income countries, which is an important factor to consider given the high cost of dental treatment, many of which are not covered by the public health systems in this region. Thus, the probability of these patients to use dental care services is lower, which may negatively impact on the OHRQoL.

It is necessary an effort of researchers and professionals working in the health systems to seek strategies that can reduce the impact of oral problems on OHRQoL. These strategies must be designed at individual, and population levels and, among them, the importance of encouraging prenatal care is highlighted. During dental prenatal care the family will receive guidance on the infant's oral health care, such as guidance on breastfeeding, healthy eating and sugar intake, counseling on non-nutritive oral habits, beginning of toothbrushing, use of fluoridated toothpastes and flossing ^29^. To date, many parents/caregivers believe in the myth that there is no need to take the infant to the dentist, since he has no teeth or teeth will be replaced by permanent ones. In Peru, for example, parents take their children to a dental appointment for the first time at the age of 4, as they do not consider deciduous teeth as important as permanent teeth ^15^. Thus, researchers, health professionals, and managers of the health system must come together so that prevention programs for oral health problems are implemented during the gestational period.

The present study has some limitations inherent to the study design, such as the impossibility of asserting a causal relationship between the child's age and impact on OHRQoL. However, it is important to emphasize the strengths, since this is a multicenter study, representative of children aged 1-3 years that uses a questionnaire with good methodological quality, cross-culturally adapted and validated for use in LAC countries ^30^. In addition, there are few studies in the literature on OHRQoL in children of this age group, most of which were carried out in Brazil and we must consider that oral health problems may present in different magnitudes in other countries in the region ^15^ ^,^ ^31^. Thus, it is important that future studies are carried out to better understand the panorama of each country so that interventions are carried out based on the needs of each population.

The use of subjective criteria is a positive point both in research and in clinical practice, since subjective measures, such as OHRQoL, aim to measure broad consequences of poor oral health ^32^. The report of parents/caregivers is essential, as they are the main decision-makers regarding their children's health care ^33^. Thus, understanding parents' perceptions of children's oral health can help in a patient-centered treatment, prioritizing care according to the family perspective, as well as the individual context in which each child is inserted ^33^. It is also worth considering that there is a direct relationship between the individual's oral health and general health and, thus, improving the quality of a patient's well-being goes beyond simply treating dental diseases and disorders ^34^.

In conclusion, multicenter epidemiological studies and national surveys should be developed to assess ORHQoL in different age groups, in order to understand the panorama of the population. These studies must use a well-defined methodology and must include standardized tools with satisfactory psychometric properties for each age group. Besides that, the countries of the Latin America region must share information about prevention and health promotion programs that are showing positive results since can contribute to improvements in OHRQoL of children from different countries.

## References

[1] Perazzo MF, Gomes MC, Neves ÉT, Martins CC, Paiva SM, Granville-Garcia AF (2017). Oral health-related quality of life and sense of coherence
regarding the use of dental services by preschool children. Int J Paediatr Dent.

[2] Perazzo MF, Serra-Negra JM, Firmino RT, Pordeus IA, Martins-Junior PA, Paiva SM (2020). Patient-centered assessments: how can they be used in dental
clinical trials?. Braz Oral Res.

[3] Paiva SM, Abreu-Placeres N, Camacho MEI, Frias AC, Tello G, Perazzo MF (2021). Dental caries experience and its impact on quality of life in
Latin American and Caribbean countries. Braz Oral Res.

[4] Borthwick-Duffy SA, Rowitz L (1992). Mental Retardation in the Year 2000. Disorders of Human Learning,
Behavior, and Communication.

[5] Felce D, Perry J (1995). Quality of life: It’s definition and measurement. Res Devl Disabil.

[6] Baiju RM, Peter E, Varghese NO, Sivaram R (2017). Oral Health and Quality of Life: Current concepts. J Clin Diagn Res.

[7] Chaffee BW, Rodrigues PH, Kramer PF, Vítolo MR, Feldens CA (2017). Oral health‐related quality‐of‐life scores differ by
socioeconomic status and caries experience. Community Dent Oral Epidemiol.

[8] Clementino LC, de Souza KSC, Castelo-Branco M, Perazzo MF, Ramos-Jorge ML, Mattos FF (2021). Top 100 most-cited oral health-related quality of life papers:
Bibliometric analysis. Community Dent Oral Epidemiol.

[9] Kramer PF, Feldens CA, Ferreira SH, Bervian J, Rodrigues PH, Peres MA (2013). Exploring the impact of oral diseases and disorders on quality of
life of preschool children. Community Dent Oral Epidemiol.

[10] Guedes RS, Piovesan C, Antunes JL, Mendes FM, Ardenghi TM (2014). Assessing individual and neighborhood social factors in child
oral health-related quality of life: a multilevel analysis. Qual Life Res.

[11] Corrêa-Faria P, Paixão-Gonçalves S, Paiva SM, Martins-Júnior PA, Vieira-Andrade RG, Marques LS, Ramos-Jorge ML (2006). Dental caries, but not malocclusion or developmental defects,
negatively impacts preschoolers' quality of life. Int J Paediatr Dent.

[12] Díaz S, Mondol M, Peñate A, Puerta G, Bönecker M, Martins Paiva S (2018). Parental perceptions of impact of oral disorders on Colombian
preschoolers' oral health-related quality of life. Acta Odontol Latinoam.

[13] Vollú AL, da Costa MDEPR, Maia LC, Fonseca-Gonçalves A (2018). Evaluation of Oral Health-Related Quality of Life to Assess
Dental Treatment in Preschool Children with Early Childhood Caries: A
Preliminary Study. J Clin Pediatr Dent.

[14] Antunes LAA, do Amaral JCN, Ornellas GD, Castilho T, Küchler EC, Antunes LS (2020). Oral health outcomes: the association of clinical and
socio-dental indicators to evaluate traumatic dental injury profile in low
income Brazilian children. Int J Burns Trauma.

[15] Pesaressi E, Villena RS, Frencken JE (2020). Dental caries and oral health-related quality of life of
3-year-olds living in Lima, Peru. Int J Paediatr Dent.

[16] Gómez MV, Toledo A, Carvajal P, Gomes SC, Costa RSA, Solanes F (2018). A multicenter study of oral health behavior among adult subjects
from three South American cities. Braz Oral Res.

[17] Varni JW, Limbers CA, Burwinkle TM (2007). How young can children reliably and validly self-report their
health-related quality of life?: an analysis of 8,591 children across age
subgroups with the PedsQL 4.0 Generic Core Scales. Health Qual Life Outcomes.

[18] Malta M, Cardoso LO, Bastos FI, Magnanini MM, Silva CM (2010). STROBE initiative: guidelines on reporting observational
studies. Rev Saude Publica.

[19] Tesch FC, Oliveira BH, Leão A (2008). Semantic equivalence of the Brazilian version of the Early
Childhood Oral Health Impact Scale. Cad Saude Publica.

[20] Scarpelli AC, Oliveira BH, Tesch FC, Leão AT, Pordeus IA, Paiva SM (2011). Psychometric properties of the Brazilian version of the Early
Childhood Oral Health Impact Scale (B-ECOHIS). BMC Oral Health.

[21] Bordoni N, Ciaravino O, Zambrano O, Villena R, Beltran-Aguilar E, Squassi A (2012). Early Childhood Oral Health Impact Scale (ECOHIS). Translation
and validation in Spanish language. Acta Odontol Latinoam.

[22] Martins-Júnior PA, Ramos-Jorge J, Paiva SM, Marques LS, Ramos-Jorge ML (2012). Validations of the Brazilian version of the Early Childhood Oral
Health Impact Scale (ECOHIS). Cad Saude Publica.

[23] López Ramos RP, García Rupaya CR, Villena-Sarmiento R, Bordoni NE (2013). Cross cultural adaptation and validation of the Early Childhood
Health Impact Scale (ECOHIS) in Peruvian preschoolers. Acta Odontol Latinoam.

[23] Ferreira MC, Ramos-Jorge ML, Marques LS, Ferreira FO (2017). Dental caries and quality of life of preschool children:
discriminant validity of the ECOHIS. Braz Oral Res.

[25] Zaror C, Atala-Acevedo C, Espinoza-Espinoza G, Muñoz-Millán P, Muñoz S, Martínez-Zapata MJ (2018). Cross-cultural adaptation and psychometric evaluation of the
early childhood oral health impact scale (ECOHIS) in Chilean
population. Health Qual Life Outcomes.

[26] Haukoos JS, Lewis RJ (2005). Advanced statistics: bootstrapping confidence intervals for
statistics with "difficult" distributions. Acad Emerg Med.

[27] Field A (2009). Descobrindo a estatística usando o SPSS.

[28] Scarpelli AC, Paiva SM, Viegas CM, Carvalho AC, Ferreira FM, Pordeus IA (2013). Oral health-related quality of life among Brazilian preschool
children. Community Dent Oral Epidemiol.

[29] American Academy of Pediatric Dentistry (AAPD) (2016). Guideline on Perinatal and Infant: Oral health
care. Pediatr Den.

[30] Paiva SM, Perazzo MF, Ortiz FR, Pordeus IA, Martins-Júnior PA (2018). How to Select a Questionnaire with a Good Methodological
Quality?. Braz Dent J.

[31] Fernandes IB, Costa DC, Coelho VS, Sá-Pinto AC, Ramos-Jorge J, Ramos-Jorge ML (2017). Association between sense of coherence and oral health-related
quality of life among toddlers. Community Dent Health.

[32] McGrath C, Broder H, Wilson-Genderson M (2004). Assessing the impact of oral health on the life quality of
children: implications for research and practice. Community Dent Oral Epidemiol.

[33] Gomes MC, Clementino MA, Pinto-Sarmento TC, Costa EM, Martins CC, Granville-Garcia AF (2015). Parental Perceptions of Oral Health Status in Preschool Children
and Associated Factors. Braz Dent J.

[34] Sousa RV, Clementino MA, Gomes MC, Martins CC, Granville-Garcia AF, Paiva SM (2014). Malocclusion and quality of life in Brazilian
preschoolers. Eur J Oral Sci.

